# Effect of Bonding Protocols on the Performance of Luting Agents Applied to CAD–CAM Composites

**DOI:** 10.3390/ma15176004

**Published:** 2022-08-31

**Authors:** Bruna Hilgemberg, Fabiana Suelen Figuerêdo de Siqueira, Andres Felipe Millan Cardenas, Josiane Loch Ribeiro, Andrés Dávila-Sánchez, Salvatore Sauro, Alessandro Dourado Loguercio, Cesar Augusto Galvao Arrais

**Affiliations:** 1 Department of Restorative Dentistry, State University of Ponta Grossa (UEPG), Ponta Grossa 840030-900, Brazil; 2 Department of Postgraduate Program in Dentistry, CEUMA University, São Luis 65075-120, Brazil; 3 Departmento de Odontología Restauradora y Materiales Dentales, Escuela de Odontología, Universidad San Francisco de Quito USFQ, Pampite y Diego de Robles, Quito 170901, Ecuador; 4 Dental Biomaterials and Minimally Invasive Dentistry, Departmento de Odontologia, Facultad de Ciencias de la Salud Universidad, CEU-Cardenal Herrera, Alfara del Patriarca, 46115 Valencia, Spain; 5 Department of Therapeutic Dentistry, I. M. Sechenov First Moscow State Medical University, 119146 Moscow, Russia

**Keywords:** universal adhesives, CAD–CAM, composite resin, resin cements, bond strength

## Abstract

This in vitro study aimed to evaluate the effect of different bonding strategies on the micro-shear bond strength (μSBS) of luting agents to CAD–CAM composites. Surface scanning electron microscopy (SEM) and spectroscopy by energy-dispersive X-ray spectroscopy (EDS) were performed to analyze the surfaces of the composite before and after bonding treatment. Three CAD–CAM composites were evaluated: Lava Ultimate restorative (LU), Brava Blocks (BR), and Vita Enamic (VE). The LU and BR surfaces were sandblasted using aluminum oxide, while the VE surfaces were etched using a 5% hydrofluoric acid gel according to the manufacturers’ recommendations. All surfaces were subjected to the following bonding strategies (n = 15): adhesive with silane and MDP (ScotchBond Universal, 3M Oral Care, St Paul, MI, USA); adhesive with MDP (Ambar Universal, FGM, Joinville, Brazil); adhesive without silane or MDP (Prime&Bond Elect, Dentsply Sirona, Charlotte, NC, USA), pure silane without MDP (Angelus, Londrina, Brazil), and pure silane with MDP (Monobond N, Ivoclar Vivadent, Schaan, Liechtenstei). Afterwards, tygons were filled with RelyX Ultimate (3M Oral Care), AllCem (FGM), or Enforce (Dentsply Sirona), which were light-cured and subjected to the μSBS test. Data were analyzed using two-way ANOVA and Bonferroni’s post hoc test (α = 0.05). Additional blocks (n = 15) were subjected to scanning electron microscopy (SEM) and energy-dispersive X-ray spectroscopy (EDS) before and after the surface treatment. The μSBS values on VE surfaces were higher than those observed on LU and BR surfaces (*p* < 0.001). Silane without MDP (Allcem) promoted the highest μSBS values, while silane with MDP (RelyX Ultimate) provided the highest values among all bonding strategies (*p* < 0.001). Enforce promoted no significant difference in μSBS values. SEM and EDS analyses detected noticeable changes to the surface morphology and composition after the surface treatment. The effectiveness of the bonding strategy may vary according not only to the CAD–CAM composite but also to resin cement/bonding agent/silane used.

## 1. Introduction

New CAD–CAM composite blocks have emerged as an option for indirect restorations [1,2,3,4]. By combining resin and ceramics, these materials present the benefits of resin composite, such as less abrasion of antagonistic teeth, and ceramics in terms of durability and color stability [3]. However, the failure of this type of material is higher compared to glass ceramics, and still little information is available about its marginal adaptation [5,6]. In this regard, because these materials are relatively new and their composition and chemical structure may differ among commercial brands, a variety of surface treatments has been proposed to improve bonding between resin cements and CAD–CAM composites [4,7].

Bonding resin cements to CAD–CAM composites relies on mechanical retention and chemical bonding [8]. Regarding mechanical retention, the effectiveness of each surface treatment depends on the composition of the CAD–CAM composite surface [9,10,11]. For instance, higher bond strength values can be achieved in vitro when CAD–CAM microfilled composite (MFR) surfaces are sandblasted with aluminum oxide (average particle size: 50 μm) [12,13]. Unlike the surface treatment recommended for MFR surfaces, in vitro studies have shown that acid etching with hydrofluoric acid followed by silane application on polymer-infiltrated ceramic (PIC) surfaces may result in higher bond strength [11]. Conversely, some studies have reported that adhesive systems should be applied to these materials after the physical treatment of the surface to increase micro-retention [12]. Therefore, there is clear evidence that these surface treatments have become the main choice to create mechanical retention when these CAD–CAM composites are used [7].

Establishing a bonding strategy that may lead to effective chemical bonding has been a key aim for many researchers [14]. Indeed, some studies showed that chemical treatment via silane application could increase the bond strength of resin cements to CAD–CAM composites, as well as the retention strength of CAD–CAM composite crowns [15], and the interfacial fracture toughness of resin cements with some CAD–CAM composites [16]. Conversely, further in vitro studies showed that silane application would have no effect on the bond strength of resin cements to CAD–CAM composite [13,17,18]. Therefore, no clear consensus on what bonding strategy would provide efficient chemical bonding on CAD–CAM composite surfaces has been achieved. 

Most recently, universal adhesive systems and/or silane containing 10-methacryloyloxydecyl dihydrogen phosphate (MDP) have been introduced into the market. Derived from tooth substrates, MDP is an acidic functional monomer, which may be able to create a bonding interaction with different materials such as metals and zirconia [19,20], and silane coupling agents promote chemical interactions with inorganic elements such as silicon in glass ceramics [12]. For this reason, products containing these compounds have been shown to provide high bond strength values to one MFR surface [12]. However, the influence of universal adhesive systems or silane agents with MDP on the bond strength of resin cements to other MFR and PIC surfaces has not yet been extensively addressed [21]. Although the effect of silane and an adhesive containing MDP and/or silane on the bond strength to lithium disilicate and zirconia ceramics has been previously evaluated [22,23], to the extent of authors’ knowledge, no studies were found evaluating these variables in CAD–CAM composites.

Therefore, the current study aimed to evaluate the effect of different bonding strategies, including the use of universal adhesive systems and silane with or without MDP, on the micro-shear bond strength (μSBS) of three dual-cured resin cements on three commercially available CAD–CAM composites. In addition, the CAD–CAM composites surfaces were also evaluated using scanning electron microscopy associated with energy-dispersive X-ray analysis (SEM/EDS). The hypothesis of this study was that the bonding strategy employed for luting CAD–CAM composites would play an important role in increasing the bond strength of resin cements.

## 2. Materials and Methods

### 2.1. Specimen Preparation

One PIC (Vita Enamic; VE, Vita Zahnfabrik, Bad Säckingen, Germany), and two MFR (Lava Ultimate; LU, 3M Oral Care, St. Paul, MN, USA; and Brava Block; BR, FGM, Joinville, SC, Brazil) were evaluated. The composition of each CAD–CAM block is displayed in Table 1. Thirty blocks of each CAD–CAM material were cut into two rectangular sections (10 × 10 × 6 mm) in a cutting machine (Isomet Buehler, Lake Bluff, IL, USA) under water-cooling, resulting in 45 specimens for μSBS test and 15 specimens for SEM/EDS of each CAD–CAM material. The specimens were embedded in polyvinyl chloride (PVC) filled with acrylic resin (Auto Clear, Dentbras, Pirassununga, SP, Brazil), leaving the top material surface exposed at a 3-mm height. Afterwards, the LU and BR surfaces were sandblasted with 50-μm aluminum oxide particles (Mega OX, Megablast, Brazil) for 15 s at 2-bar pressure and a distance of 4 mm [24], while the VE specimens were etched with 5% hydrofluoric acid (Vita Ceramics Ethc, Bern, Switzerland) for 60 s and thoroughly rinsed with water spray for 30 s, according to the manufacturers’ recommendations. All specimens were ultrasonically cleaned (Cristofoli Ultrasonic Cleaner, 2008, Campo Mourão, PR, Brazil) for 480 s to remove impurities and finally air-dried.

**Figure 1:**
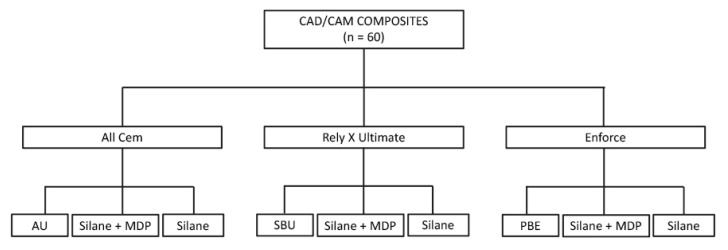
Experimental design carried out for this study.

Afterwards, the specimens were randomly assigned to each bonding strategy, as displayed in Figure 1. In summary, the surfaces were subjected to the following bonding strategies: the adhesive and resin cement from the same manufacturer (ScotchBond Universal/RelyX Ultimate, 3M Oral Care, St. Paul, MN, USA; Ambar Universal/All Cem, FGM Dental Group, Joinville, SC, Brazil; Prime & Bond Elect/Enforce, Dentsply Sirona, Charlotte, USA), silane without MDP (Silano, Angelus, Londrina, PR, Brazil), and silane with MDP (Monobond N, Ivoclar Vivadent, Schaan, Liechtenstein). More details of the products and their application modes are described in Table 2.

### 2.2. μSBS Test

**Figure 2:**
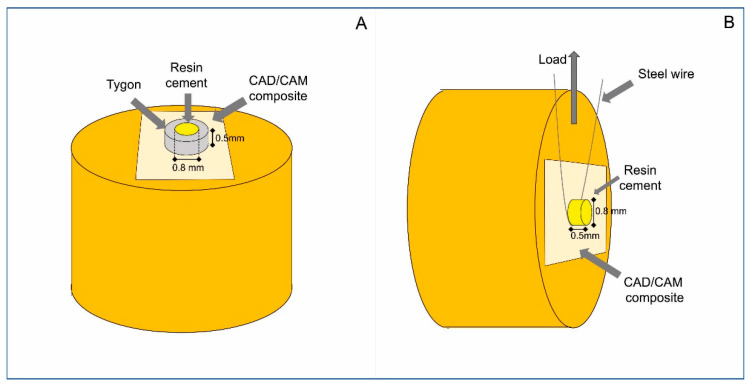
Schematic drawing of the SBS test used in the current study. (**A**) Specimen preparation on the CAD–CAM composite surface; (**B**) Specimen positioned for the SBS test.

After the bonding procedures were performed on the surfaces of the tested composites, eight cylindrical transparent tygon-type polyethylene tubes (Tygon Medical Tubing Formulations 54-HL, Sai Gobain Performance Plastics, Akron, OH, USA), with an internal diameter of 0.8 mm and 0.5 mm height, were positioned on each treated surface. The dual-cured resin cements RelyX Ultimate (3M Oral Care), AllCem (FGM) and Enforce (Dentsply Sirona) were manipulated and carefully inserted into each tygon tube using a #5 explorer (SSwhite/Duflex, Rio de Janeiro, RJ, Brazil) to fill its total internal volume. A transparent Mylar strip was placed over the tygon tube and gently pressed into place. The resin cement was then photoactivated for 20 s using an LED light-curing (Radii-Cal, Irradiance: 1200 mW/cm^2^, SDI, Victoria, Australia). The irradiance was constantly checked with a radiometer (Demetron L.E.D. Radiometer, Kerr Sybron, Middleton, WI, USA). These procedures were performed under magnifying glasses. After storage in distilled water for 24 h at 37 °C, the specimens had the tygon tubes carefully removed with an No 11 scalpel blade. Each specimen was then examined under a stereomicroscope (Olympus SZ40, Shinjuku-ku, Tokyo, Japan) at a 10× magnification. The presence of porosities or cracks in the bonding interface was evaluated, and the defective cylinders were discarded. The specimens were then attached to a micro-shear testing device (Odeme Biotechnology, Joaçaba, SC, Brazil), and an orthodontic steel wire (0.2 mm diameter) was positioned to surround the bottom of each resin cement cylinder. The device was aligned to pull the resin cement interface–CAD–CAM composite perpendicularly to the center of the load cell (Figure 2). The specimens were then tested on a universal testing machine (Kratos IKCL 3-USB, Kratos Equipamentos Industriais, Cotia, SP, Brazil) at a 1 mm/min rate until failure. After testing, the specimens were examined under an optical microscope (SZH-131, Olympus; Tokyo, Japan) with a magnification of 10× to determine the fracture pattern, which was classified as cohesive within the resin cement (CRC), cohesive within the CAD–CAM composite (CCC), and adhesive/mixed (A/M fracture at the CAD–CAM composite surface that included cohesive fracture of neighboring substrates).

### 2.3. Surface Scanning Electron Microscopy (SEM) and Spectroscopy by Energy-Dispersive X-ray Spectroscopy (EDS)

Fifteen additional blocks of each CAD–CAM material that were not previously used in the μSBS test had their surfaces cleaned with 70% ethanol, air-dried, and treated either according to the manufacturer instructions or according to the experimental protocol, and then ultrasonically cleaned (Cristofoli Ultrasonic Cleaner, 2008, Campo Mourão, PR, Brazil) for 480 s and cleaned with alcohol 70%, air-dried, and positioned on a metallic stub. All specimens were dried and dehydrated in a desiccator for 12 h and were then sputter-coated with a gold/palladium alloy (SCD 050, Balzers, Schaan, Liechtenstein). The treated surfaces were examined under a scanning electron microscope (MIRA3 LM, Tescan Orsay Holding, Warrendale, PA, USA). Three representative photomicrographs of each surface were obtained at a 2500× magnification, and the chemical elements present on the CAD–CAM surfaces before and after the surface treatments were analyzed by energy-dispersive X-ray spectroscopy (EDS) coupled to the SEM.

### 2.4. Statistical Analysis

The μSBS data were subjected to the Shapiro–Wilk normality test to verify normality. Homogeneity of variances was checked using Levenes’ test. Once the data had passed, statistical analysis was performed using two-way ANOVA and Bonferroni’s post hoc test (α = 0.05), with the “CAD–CAM composites” and “bonding strategy” as independent variables. No comparisons were made among resin cements. Post hoc power analysis was performed at a pre-set alpha of 0.05. All statistical analyses were performed using commercial statistical software (Statistics 19, SPSS Inc., IBM Company, Armonk, NY, USA).

## 3. Results

### 3.1. μSBS Test

The mean values of μSBS (MPa) and standard deviations of each experimental group (LU, BR, and VE) are shown in Table 3, Table 4 and Table 5. Based on the post hoc power analysis, the number of specimens evaluated in the μSBS test generated a statistical power above 90% (α = 5%). Overall, the μSBS values on VE surfaces were higher than those observed on LU and BR surfaces (*p* < 0.001), regardless of resin cement/bonding strategy.

For AllCem resin cement, two-way ANOVA detected a statistical significance for the variables “CAD–CAM composites” (*p* < 0.01) and “bonding strategy” (*p* = 0.017). The use of silane without MPD promoted the highest μSBS values, while the application of silane with MDP presented the lowest values.

When using Rely X Ultimate resin cement, two-way ANOVA detected a statistical significance for “CAD–CAM composites” (*p* < 0.01), “bonding strategy” (*p* = 0.02), as well as for the interaction between factors (*p* = 0.02). The bonding strategy only influenced the μSBS values in the VE groups, as silane with MDP promoted the highest values.

When Enforce was evaluated, there was a statistical significance only for the variable “CAD–CAM composites” (*p* < 0.01). No significant difference was noted in the μSBS values when different bonding strategies were used. The highest μSBS values were observed on the VE surface, while the lowest values were noted on the BR surface, regardless of the bonding strategy.

### 3.2. Fracture Pattern (PF)

**Figure 3:**
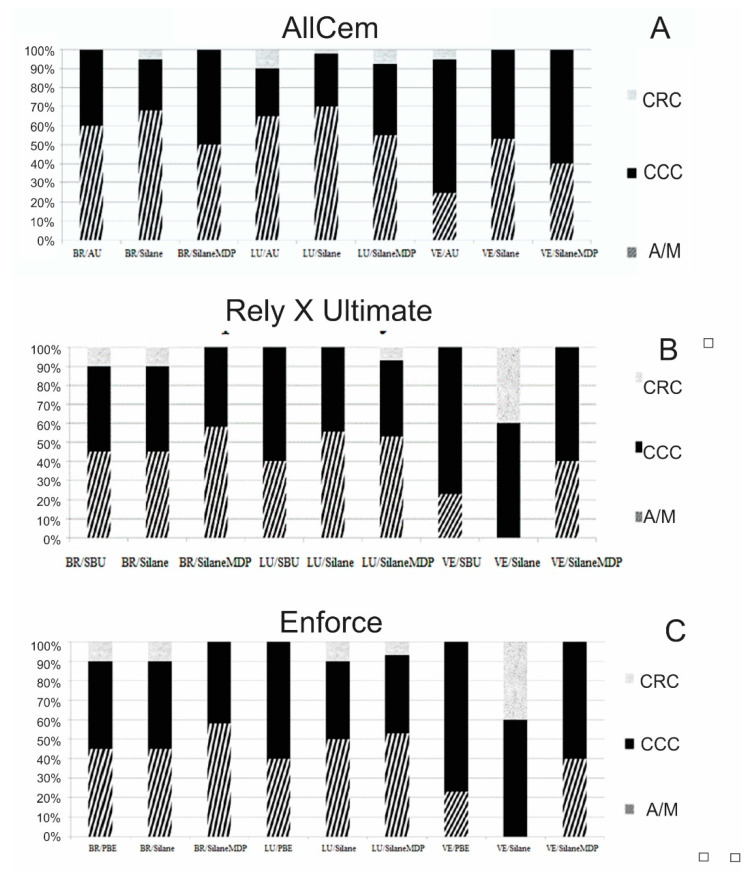
Distribution of failure pattern in the experimental groups using AllCem (**A**), Rely X Ultimate (**B**), and Enforce (**C**).

The fracture pattern distribution for each group (LU, BR and VE) and for the resin cements are shown in Figure 3. Most groups presented failures predominantly located at the cement–composite interface. In contrast, the cohesive failure represented the lowest percentage when BR and LU composites were evaluated. A small percentage of cohesive fracture within resin cement was noted for most groups. When the VE surface was assessed, the percentage of cohesive fractures within the CAD–CAM composite was similar to or higher than the percentage of adhesive fractures.

### 3.3. Surface Scanning Electron Microscopy (SEM)

**Figure 4:**
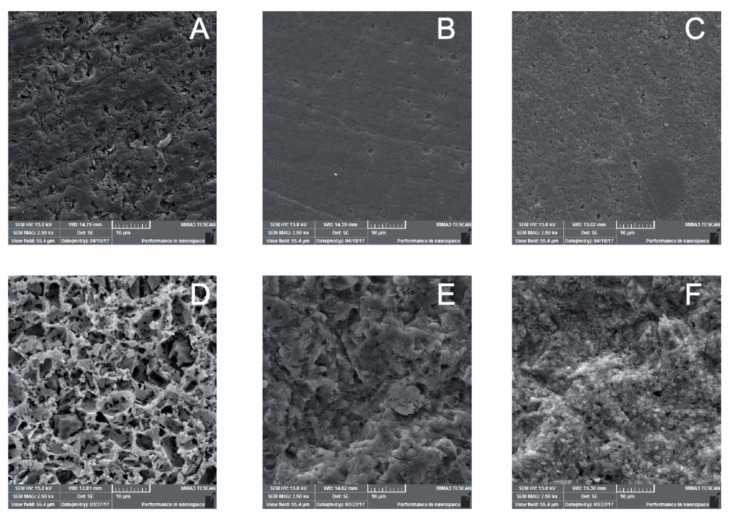
Representative SEM photomicrography of untreated (**A**–**C**) and treated (**D**–**F**) surfaces of VE, LU, and BR CAD–CAM composites (2500×).

The SEM representative photomicrographs from each experimental group are shown in Figure 4. After sandblasting, in the Lava Ultimate and Brava surfaces, an apparently more irregular surface was noted (Figure 4E,F), in addition to the exposure of the polymeric portion and the vitreous portion of the ceramics in Brava Block surface (Figure 4F). However, no porosities were noted.

Among the CAD–CAM composites, the VE composite showed a more significant surface change after the surface treatment with hydrofluoric acid. Degradation of the vitreous matrix was noticed, exposing the organic matrix and causing irregularities and micro porosities as a consequence (Figure 4D).

### 3.4. Energy-Dispersive X-ray Spectroscopy (EDS)

The EDS analysis of untreated and treated surfaces are presented in Table 6 and Table 7, respectively. LU surface was the only material presenting zirconia in its composition and the highest vitreous content (silica). After surface treatment, the percentage of silica and zirconia decreased on that surface. Apparently, the VE surface showed more aluminum in its composition than the other CAD–CAM composites. After surface treatment, no noticeable changes to the composition of BR and VE surfaces were noted.

## 4. Discussion

In the current study, only one type of resin cement (AllCem), silane without MDP, promoted the highest μSBS values regardless of CAD–CAM composite. On the other hand, the highest values were noted when silane with MDP was applied prior to the use of RelyX Ultimate on the VE surface, while no significant differences in the μSBS values were observed when that resin cement was applied to the other CAD–CAM composites, regardless of the bonding strategy. Likewise, the bonding strategy had no influence on the μSBS values when Enforce was used, regardless of CAD–CAM composite. Therefore, the influence of the bonding strategy on μSBS values was resin cement/bonding agent-dependent rather than the CAD–CAM composite used. Thus, the hypothesis that established that the bonding strategy would play an important role in increasing the bond strength of CAD–CAM composites was partially accepted.

A significant difference in the μSBS values was noticed among the CAD–CAM composites regardless of the bonding strategy and cement/bonding agent. More specifically, among the CAD–CAM composites evaluated in the current study, the highest μSBS values were obtained with the VE composite, regardless of the bonding strategy. It is important to highlight that the inorganic portion of Vita Enamic is composed of feldspar ceramics [1,7] as confirmed by the EDS analysis. For this reason, that material can be acid-etched using hydrofluoric acid. On the other hand, Lava Ultimate has its inorganic portion composed of nanoceramics reinforced by zirconia, which provides greater resistance to the material, but cannot be acid-etched by common dental acids [25]. For this reason, there is a clear distinction between the manufacturer instructions regarding the protocol of surface treatment. For instance, the recommended surface treatment of Lava Ultimate and Brava Block is sandblasting, while etching with hydrofluoric acid is recommended for materials such as Vita Enamic. Therefore, on VE surfaces, hydrofluoric acid preferentially dissolves the glassy or crystalline portion as it reacts with the silica present in the glassy matrix [12,20,26]. It also removes the organic portion (polymers), producing porosities of up to 10 μm depth that were not seen on the other surfaces, resulting in a microstructure that favors the bond strength of the bonding agents/resin cement to the surface. Such porosities resulting from acid etching were clearly seen in the SEM analysis (Figure 4).

In the current study, the use of silane with MDP had no positive effect on the μSBS values in the MFR composite. The MDP molecule has a phosphoric acid group at one end and a vinyl group at the other end of the molecule [12]. The phosphoric acid group can chemically bond to zirconia and silica [27], which increases the bonding to surfaces with zirconia or barium, such as Lava Ultimate and Brava Block composites, respectively [12]. However, according to Stawarczyk et al. [28], as the silica and zirconia particles from LU composite and the barium glass particles from BR composite are pre-silanized, it must be questioned whether the MDP’s pathway to interact with silica may have been compromised. This fact would not only explain why MDP had no impact on the μSBS values of LU composite but also why the bonding agent and silane with MDP did not promote the highest μSBS values when applied using AllCem. In addition, based on the EDS analysis, only a small concentration of zirconia (12.1%) was present on the LU surface after sandblasting, so the total area available for chemical bonding between zirconia and MDP was not high enough to significantly contribute to μSBS values.

Although no significant difference in the μSBS values was observed when SBU/Rely X Ultimate was applied to the RMF surfaces regardless of the bonding strategy, SBU and the silane without MDP promoted significantly lower μSBS values than did the silane with MDP on the VE surface. According to Chen L et al., 2013 [29], the contact of Bis GMA also present in the SBU composition with silane can prevent the action with the hydroxyl groups present on the treated surface. In addition, it has been shown that the efficacy of the silane contained in the SBU composition may be compromised over time due to the fact that it is in contact with the acidic monomer MDP [30,31], which leads the silanol groups to premature reactions of hydrolysis and dehydration condensation, forming oligomers that are not able to bind to the material. That would also help explain the lower μSBS values when silane with MDP was applied prior to the use of AllCem. Conversely, one could state that lower μSBS values should be expected on the CAD–CAM composites when Enforce was applied along with PBE. However, it is worth noticing that PBE has penta-p molecules in its composition, which has been shown to improve the bond strength to CAD–CAM composites.

In the current study, the most predominant failure pattern observed was adhesive/mixture or cohesive within the CAD–CAM composite depending on the CAD–CAM material, surface treatment, and cementing system. Apparently, an adhesive/mixed failure pattern was predominantly seen when the system Prime & Bond Elect/Enforce was used. On the contrary, an apparently higher percentage of cohesive failure within the VE composite was seen in most groups. These findings are similar to those shown by El-Damanhoury and Gaintantzopoulou [26], who stated that this outcome could be attributed to the micro-interlocking created by hydrofluoric acid, which increases the bond strength to higher values than the cohesive strength of that CAD–CAM composite.

The EDS analysis is considered a valuable tool for the chemical characterization of a wide range of materials. In this sense, given the principles of adhesion between materials (e.g., surface roughness, wettability and chemical interaction between materials of different composition), a comprehensive knowledge regarding substrates composition may bring to light specific and predictable protocols based on substrates chemistry. In this regard, the EDS analysis of the CAD–CAM composite surfaces confirmed that Lava Ultimate (LU) was the only restorative material that presented a relevant percentage of zirconia (Zr) and the only one that showed a higher amount of silica. Vita Enamic (VE), on the other hand, had a higher amount of aluminum, related to the presence of feldspar ceramic in its composition. For the CAD–CAM LU composite, a percentage of silica, zirconia, and oxygen was seen, featuring a ceramic reinforced by zirconia. For the CAD–CAM VE composite, aluminum, potassium, sodium, silica, and oxygen were observed in the analysis, confirming that this material consists of feldspar ceramic. Most importantly, the analysis performed after the surface treatments revealed that the percentage of the chemical elements remained constant on BR and VE surfaces compared to the analysis performed before the surface treatments. Curiously, on the LU surface, the detected percentage of silica and zirconia was apparently lower than that observed before the treatment. In addition, a small percentage of aluminum appeared, which may be related to the treatment with aluminum oxide once no aluminum was detected prior to the surface treatment. Therefore, the impact of the residual aluminum on the bond strength to RMF surfaces deserves further investigation. In addition, the fact that the LU presented different values after the surface treatment may be related to the empty spaces and the irregular, rougher surface created by sandblasting, as also observed by other authors [13,18]. However, it should be mentioned that sandblasting may damage the CAD–CAM surface depending on the composition of the CAD–CAM material [32,33]. The findings obtained in our study regarding EDS analysis may not be conclusive, as in several scenarios, no differences in the bonding performance were observed between each material using different luting agents. In this regard, the chemical interaction between CAD–CAM composites and the luting strategy remains unclear to the authors. However, it can be speculated that other inherent physical and surface properties of both substrate, primers and luting agent/adhesive, such as surface energy obtained after substrate conditioning, surface tension of the luting agent and the topography of the material, may have a foremost role in the bonding and performance of these specific type of materials.

Although the current in vitro study showed the importance of different bonding strategies on short-term μSBS values of some resin cements on RFM and PIC surfaces, further studies are required to assess the long-term effects of the bonding strategies evaluated in the study. In addition, since this study focused only on the interface created between adhesive systems and CAD–CAM composites, studies involving the dental substrate are also required to provide a better understanding of such a complex interaction between bonding systems and tooth substrate.

## 5. Conclusions

Within the limitations of this study, it can be concluded that the effectiveness of the bonding strategy may depend mainly on the resin cements/bonding agents employed rather than on the CAD–CAM composite. Moreover, polymer-infiltrated ceramics such as Vita Enamic (VE) may present favor greater bond strength values regardless of the bonding strategy.

## Figures and Tables

**Table 1:** Description of materials, manufacturers, compositions, and indications of CAD–CAM composites.

Trademarks Experimental Groups	Composition	Indication
Vita Enamic, Vita Zahnfabrik, Bad Säckingen, Alemanha (VE)	Feldspar ceramics reinforced with aluminum oxide + polymer (UDMA and TEGMA).	Single-sided implant tooth crowns, inlays, onlays and veneers.
Lava Ultimate 3M Oral Care, St. Paul, MN, USA (LU)	Silicon nanoparticles, zirconia nanoparticles, nanoclusters, silicon union and a resinous matrix.	Permanent unit crowns about implant, facets, inlays and onlays.
Brava Block FGM, Joinville, SC, Brazil (BR)	65 to 80% silanized barium glass, Bis EMA, Bis GMA, Dimethylaminobenzoate and camphorquinone.	Inlays, onlays and laminates.

UDMA: urethane dimethacrylate; TEGMA: triethylene glycol dimethacrylate; Bis EMA: bisphenol A ethoxylated dimethacrylate; Bis GMA: bisphenol A-glycidyl methacrylate.

**Table 2:** Description of the materials, manufacturers, compositions, application method, and indication of the materials used in this study.

Hydrofluoric acid 5% (Vita ceramics etch)	1 mL of acid VITA CERAMICS ETCH contains 0.047 g of hydrofluoric acid.	1—Apply using a microbrush on the surface of restoration for 60 s; 2—Wash abundantly; 3—Apply jet air.
Monobond N Ivoclar Vivadent (silane with MDP)	Alcoholic solution of methacrylate silane, ethanol, 10-MDP, and sulfide methacrylate.	1—Apply a drop with the aid of a microbrush; 2—Let it react for 60 s; 3—Apply air jet strongly.
Silane Angelus (Silane without MDP)	Methylene, oxygen and oxygen, silicon, ethanol, hydroxyl.	1—Apply on the surface; 2—Stand by 60 s; 3—Apply jet air.
Single Bond Universal (SBU) 3M Oral Care	MDP, Dimetacrilate resins, HEMA, VitrebondTM copolymer. Filling particles, ethanol, water, initiators, Silane.	1—Apply an active layer on the surface; 2—Leave solvent to evaporate for 5 s; 3—Jet air for 5 s; 4—Light cure for 20 s.
Prime & Bond Elect (PBE) Dentsply Sirona	Mono, di- and trimetacrylate resin, PENTA, diacetone, phosphine organic, stabilizers, ketylamino fluoride, and acetone, water, acetone, catalyst, photoinitiators.	1—Mix one drop of each vial; 2—Apply a layer over the surface; 3—Allow to evaporate for 20 s; 4—Apply air jet for 5 s; 5—Light cure for 20 s.
Ambar Universal (AU) FGM	Methacrylic monomers (MDP and UDMA), photoinitiators, co-conspirators and stabilizers, in addition to inert load (nanoparticles of silica) and vehicle (ethanol).	1—Actively apply two layers of adhesive on the surface; 2—Jet air for 10 s between the layers; 3—Light cure for 20 s.
Rely X Ultimate 3M Oral Care	Glass powder treated with methyl propanoic silane, hydroxymethyl Ester, reaction products with hydroxy propanediol dimethacrylate and phosphorus oxide, TEGDMA, silane-treated silica, glass borosilicate, sodium persulphate, peroxy-trimethylhexanoate-butyl and monohydrated copper acetate.	1—Mix the two folders; 2—Apply on the surface; 3—Light cure for 40 s.
Enforce Dentsply Dentsply Sirona	Base Paste: Glass from Boron, aluminum silicate and Silanized barium, pyrolytic silica Silanized, Bis GMA, BDMA, BHT, Camphorquinone, TEGDMA, Mineral pigments, EDAB. Catalytic Paste: Glass Boron, aluminum silicate and silanized barium, pyrolytic silica silanized, BisGMA, BDMA, BHT, TEGDMA, stains, Benzoyl minerals and peroxide.	1—Mix the two folders; 2—Apply on the surface; 3—Light cure for 40 s.
AllCem FGM	Methacrylic monomers (TEGDMA and HEMA); camphorquinone; co-initiatorsand microparticles of barium glass.	1—Mix the two folders; 2—Apply on the surface; 3—Light cure for 40 s.

Abbreviations: HEMA: methacrylate of hydroxyethyl; MDP: methacryloyloxydecyl dihydrogen phosphate; BisGMA: bisphenol glycidyl methacrylate; PENTA: Dipentaerythritol monophosphate penta acrylate; TEGDMA: Triethylene glycyl methacrylate; BDMA: Butanediol dimethacrylate; BHT: Butyl ethyl phenol; EDAB: ethyl 4-dimethylaminobenzoate.

**Table 3:** Means and standard deviations of bond strength values (MPa) by micro-shear test with AllCem cement for the experimental groups.

	AllCem			
	Adhesive with MDP and without Silane (AU)	Silane without MDP	Silane with MDP	Average
Brava Block (BR)	21.23 ± 1.5	22.15 ± 2.3	21.11 ± 2.3	21.50 C
Lava Ultimate (LU)	24.87 ± 2.0	24.88 ± 2.1	22.68 ± 1.9	24.14 B
Vita Enamic (VE)	25.88 ± 3.2	28.50 ± 2.7	25.89 ± 3.3	26.76 A
Average	23.99 ab	25.18 a	23.23 b	

Means followed by different letters (uppercase letters within column; lower case letters within row) are significantly different (pre-set α = 0.05).

**Table 4:** Means and standard deviations of bond strength values (MPa) by micro-shear test with Rely X Ultimate cement for the experimental groups.

	Rely X Ultimate			
	Adhesive with Silane and MDP (SBU)	Silane without MDP	Silane with MDP	Average
Brava Block (BR)	19.16 ± 2.3 a	20.21 ± 2.4 a	20.05 ± 1.8 a	19.80 C
Lava Ultimate (LU)	21.65 ± 2.2 a	23.16 ± 1.9 a	23.26 ± 2.7 a	22.70 B
Vita Enamic (VE)	27.50 ± 3.6 b	25.9 ± 2.0 b	30.48 ± 3.2 a	27.96 A

Means followed by different letters (uppercase letters within column; lower case letters within row) are significantly different (pre-set α = 0.05).

**Table 5:** Averages and standard deviations of bond strength values (MPa) by micro-shear test with Enforce cement for the experimental groups.

	Enforce			
	Adhesive without MDP and Silane (PBE)	Silane without MDP	Silane with MDP	Average
Brava Block (BR)	20.99 ± 1.7	20.58 ± 1.8	20.33 ± 2.4	20.63 C
Lava Ultimate (LU)	21.87 ± 1.9	24.12 ± 2.2	22.16 ± 2.2	22.72 B
Vita Enamic (VE)	26.99 ± 2.5	27.36 ± 3.2	25.12 ± 2.8	26.49 A
Average	23.28 a	24.02 a	22.54 a	

Means followed by different letters (uppercase letters within column; lower case letters within row) are significantly different (pre-set α = 0.05).

**Table 6:** EDS analysis of untreated surfaces.

	Percentage of Chemical Elements (%)						
CAD–CAM Composites	K	Al	Ba	Na	Si	Zr	O
Brava Block (BR)	0	5.53	22.27	0	30.23	0	41.96
Lava Ultimate (LU)	0	0	0	0	36.57	16.11	47.32
Vita Enamic (VE)	5.14	11.8	0	5.65	29.87	0	47.54

**Table 7:** EDS analysis of treated surfaces.

	Percentage of Chemical Elements (%)						
CAD–CAM Composites	K	Al	Ba	Na	Si	Zr	O
Brava Block (BR)	0	5.18	24.15	0	29.56	0	41.10
Lava Ultimate (LU)	0	1.82	0	0	23.15	12.71	62.31
Vita Enamic (VE)	5.76	11.41	0	4.16	30.81	0	47.87

## Data Availability

Not applicable.
